# SlMYB76, an SlANS-Repressing R2R3-MYB Transcription Factor, Regulates Anthocyanin Accumulation in ‘Black Pearl’ Tomato (*Solanum lycopersicum*)

**DOI:** 10.3390/genes16111291

**Published:** 2025-10-30

**Authors:** Shuang Ma, Zedi Duan, Xiaojuan Yin, Lijing Chen

**Affiliations:** 1 College of Horticulture, Shenyang Agricultural University, Shenyang 110161, China; mashuang1326@126.com (S.M.);; 2 Shenyang Institute of Technology, College of Life Engineering, Shenfu Reform and Innovation Demonstration Zone, Shenyang 113122, China; yinxiaojuan43@126.com

**Keywords:** *Solanum lycopersicum*, anthocyanin, *SlMYB76*, MBW complex, negative regulation

## Abstract

**Background/Objectives**: Anthocyanins, water-soluble flavonoid pigments with critical roles in plant stress resistance, are not naturally accumulated in cultivated tomato (*Solanum lycopersicum*) due to an incomplete flavonoid metabolism pathway. In contrast, ‘Black Pearl’ tomato exhibits distinct peel color transitions (from indigo rose to deep purple–red) during ripening, making it an ideal model for investigating the regulatory mechanisms of anthocyanin synthesis. A comprehensive strategy was employed to elucidate these mechanisms, involving transcriptomic (Illumina HiSeq), metabolomic (UPLC-MS/MS), and functional analyses of the ‘Black Pearl’ tomato peel across four developmental stages: mature green (S1), coloring (S2), purple immature (S3), and fully ripened (S4). **Results**: Transcriptome profiling identified 597 core differentially expressed genes (DEGs) associated with anthocyanin accumulation. Temporal analysis indicated that structural genes and activators peaked at S3, whereas repressive MYBs, including *SlMYB76* which peaked at S2, exhibited staged expression. In parallel, metabolomic analysis identified 36 metabolites, with cyanidin and pelargonidin derivatives being characterized as the principal pigments. Functionally, *SlMYB76* was confirmed to be a negative regulator, as its transient overexpression reduced anthocyanin content and downregulated *SlANS*. Mechanistically, direct binding and repression of the *SlANS* promoter by *SlMYB76* were confirmed through yeast one-hybrid and dual-luciferase assays. Furthermore, its physical interaction with the bHLH factor SlJAF13 in the nucleus was demonstrated by Y2H, BiFC, LCI, and Co-IP, supporting the formation of a repressive complex that co-regulates *SlANS*. **Conclusions**: A novel SlMYB76-SlJAF13-SlANS regulatory module controlling anthocyanin accumulation in the peel of ‘Black Pearl’ tomato was identified. This discovery enhances the current understanding of the tomato flavonoid regulatory network and provides strategic targets for the genetic improvement of fruit color and anthocyanin content through molecular breeding.

## 1. Introduction

Anthocyanins, the water-soluble flavonoids responsible for most red-to-blue pigmentation in plants, are of particular interest in tomato due to their absence in commercial varieties and potential health benefits. Beyond their aesthetic value, they exhibit strong antioxidant properties, enabling them to effectively scavenge free radicals and reactive oxygen species (ROS) under environmental stress [1]. As the largest subgroup of flavonoids, anthocyanin biosynthesis begins with phenylalanine and proceeds through a cascade of reactions mediated by dedicated structural enzymes [2]. Following synthesis, anthocyanins are transported to vacuoles for storage [3]. Subsequently, anthocyanins are often modified by enzymes such as anthocyanin reductase (ANR) and leucoanthocyanidin reductase (LAR) in fiber cells, yielding a diverse spectrum of stable pigments [4].

Anthocyanin synthesis is intricately regulated by various transcription factors. Key regulators include HY5, BBX, WRKY, EIL1, bZIP, and the MYB-bHLH-WD40 (MBW) ternary complex, which control the transcription of structural genes in this pathway [5,6,7]. In tomato, as in other plants, R2R3-MYB transcription factors have emerged as master switches controlling the flux through the anthocyanin pathway. Overexpression of activators like *MdMYB24L*, *PaMYB10*, *AcMYB123*, *LcMYB5*, and *FcMYB123* leads to increased anthocyanin content in tissues [8,9,10]. Conversely, repressors such as *StMYB44* inhibit anthocyanin biosynthesis in potato tuber flesh under high-temperature conditions [11]. This repressive function often involves competition for bHLH partners, as demonstrated by *PpMYB18* in peach, which fine-tunes anthocyanin accumulation by competing with activators [12]. Additionally, *FtMYB3* in tartary buckwheat root tissue negatively regulates anthocyanin biosynthesis by downregulating key structural genes under abiotic stress [13]. Collectively, transcription factors, especially MYBs, are pivotal in fine-tuning anthocyanin synthesis, contributing to the diverse pigmentation patterns observed across plant tissues and environmental conditions.

Tomato is a widely cultivated horticultural crop known for its incomplete flavonoid metabolism pathway. Although cultivated tomato fruits contain flavonols like quercetin, kaempferol, and naringenin, they do not naturally accumulate anthocyanins [14,15]. For instance, the transfer of *CHS* from petunia to tomato increased flavonol levels but did not result in anthocyanin accumulation in fruits [16]. The successful engineering of purple tomatoes has been demonstrated through the fruit-specific, heterologous expression of transcription factor pairs, notably the bHLH factor Delila (Del) and the MYB factor Rosea1 (Ros1) [17]. In wild tomatoes, increased fruit anthocyanin levels are observed in natural variants like *Anthocyanin fruit* (*Aft*) and *atroviolacea* (*atv*). The *Aft* locus is associated with the candidate gene *SlAN2-*like (Solyc10g086290), which is defined as an encoder of a R2R3-MYB transcription factor. The expression of anthocyanin biosynthetic genes is significantly downregulated by mutations in this gene. Additionally, repression is mediated by the SlAN2-like target, SlMYBATV. A physical interaction with SlJAF13 is also exhibited by the SlMYBATV protein. Yeast one-hybrid and dual-luciferase assays established that Aft protein directly binds and activates *SlMYBATV* promoter, thereby defining a key regulatory pathway for this process [18].

To elucidate the regulatory mechanisms controlling peel color transition, we investigated the ‘Black Pearl’ tomato, a unique variety whose peel transitions from a striking indigo-rose at near maturity to a purple–red upon full ripening. To this end, dynamic shifts in genetic and metabolic profiles across key developmental stages were characterized, with a specific focus placed on the transcription factor SlMYB76. Through integrated in vivo and in vitro experimental approaches, the specific role of SlMYB76 in controlling anthocyanin production was delineated. These findings provide novel insights into tomato flavonoid metabolism and identify key genetic targets, thereby contributing to the development of breeding strategies for anthocyanin-enriched varieties.

## 2. Materials and Methods

### 2.1. Plant Materials and Growth Conditions

Studies were conducted on ‘Black pearl’ tomato (*S. lycopersicum*) and tobacco (*Nicotiana benthamiana*) plants, which were maintained under controlled greenhouse settings (25 ± 2 °C, 60% RH, 16/8 h light/dark cycle). Tomato fruit ripening was categorized into four distinct stages based on peel coloration and anthocyanin accumulation dynamics: S1 (mature green)—fruits develop to maturity under shaded conditions without pigment accumulation; S2 (coloring)—anthocyanin accumulation begins; S3 (purple immature)—anthocyanin levels peak; S4 (red ripening)—anthocyanin content declines. For each sample, Pericarps from three fresh tomatoes were pooled, frozen in liquid nitrogen and maintained at −80 °C for subsequent analysis.

### 2.2. Transcriptome Data Source and Bioinformatic Analysis

Total RNA was isolated from ‘Black Pearl’ tomato peels. Sequencing libraries were prepared from oligo(dT)—enriched mRNA by fragmentation, reverse transcription with M-MLV, second-strand synthesis, and then end repair, A-tailing, and adapter ligation.

Raw sequencing reads were processed through the tomato reference genome (SL4.0) using the STAR aligner for subsequent transcript quantification and normalization. Transcript abundance for annotated genes was quantified and normalized. Genes were defined as differentially expressed based on a *p*-value < 0.05 and an absolute |log_2_ Fold Change| ≥ 1.

### 2.3. Quantitative Real-Time PCR Analysis

qRT-PCR assays were conducted in 10 μL reactions comprising: 1 μL cDNA template, 5 μL MonAmp™ ChemoHS qPCR Mix(Monadbiotech, Suzhou, China ), 0.3 μL each forward and reverse primer (Supplemental Table S1), and 3.4 μL nuclease-free water. Relative expression levels were normalized to *actin* and calculated via the 2^−ΔΔCt^method [19].

### 2.4. Metabolomic Analysis

For metabolomic analysis, lyophilized and ground peel powder (100 mg) was extracted overnight at 4 °C with 1.0 mL of 70% aqueous methanol, followed by centrifugation, filtration of the supernatant through a 0.22 μm membrane, and subsequent LC-MS/MS analysis. Chromatographic separation was carried out on the UPLC-MS/MS system using a Waters ACQUITY UPLC HSS T3 C18(Waters Corporation, Milford, MA, USA) column (1.8 μm, 2.1 × 100 mm) at 40 °C.

### 2.5. Determination of Anthocyanin Content

A modification was employed for the quantification of anthocyanins [20]. The key alteration was in the extraction: 0.1 g of tomato peel powder was incubated with 600 μL of acidified methanol (1% HCl) at 4 °C overnight. Subsequent steps for chlorophyll removal and spectrophotometric calculation (A_530_ − 0.33 × A_657_) were performed as standard. Three biological replicates were analyzed.

### 2.6. Dual-Luciferase Assay

The coding sequence (CDS) of *SlMYB76* was cloned into the pGreen II 62-SK vector at the *EcoR*I restriction site. The upstream 2000 bp promoter sequence of the *SlANS* was cloned into the pGreen-0800-LUC vector at the *Sma*I restriction site. The Effector and Reporter vectors were transformed into *Agrobacterium* GV3101 competent cells. To prepare the bacterial suspension, 1 mL of overnight cultured *Agrobacterium* was transferred into 25 mL of liquid LB medium. 2 μL of 100 mM acetosyringone and 100 μL of 0.5 M MES were added to the culture. To induce virulence, the culture was amended with 200 μM acetosyringone and 20 mM MES. The culture was incubated at 28 °C to an OD_600_ of 1.0. Harvested by centrifugation (4000 rpm, 10 min, room temperature), the bacterial cells were resuspended in 10 mM MgCl_2_ to an OD_600_ of 1.0. This suspension was supplemented with 200 μM acetosyringone and incubated for at least 3 h.

For infiltration, healthy tobacco leaves in the growing stage were selected. Small holes were made on the underside of the leaves using a needle. The infiltration solution, prepared by mixing 1 mL of pGreen II 0800-LUC and 4 mL of pGreen II 62-SK, was loaded into a 5 mL syringe. The liquid was injected into the tobacco leaves through the lower epidermis using thumb pressure on the syringe plunger. Successfully infiltrated tobacco leaves would become wet. After 72 h of infiltration, samples were taken, and the luciferase activity was analyzed using a luminometer with the dual-luciferase assay kit from Promega. The fluorescence value of the target gene plasmid was calculated as the ratio of the fluorescence value of the reference plasmid (F/R ratio) based on the fluorescence values obtained from the dual-reporter system.

### 2.7. Yeast One-Hybrid Assay

The coding sequence (CDS) of *SlMYB76* and the promoter fragment of *SlANS* were cloned into the pGADT7 and pABAi vectors using *EcoR*I/*BamH*I and *Xho*I/*Sma*I restriction sites, respectively. The bait strain was constructed by cloning the *SlANS* promoter into pABAi (*Xho*I/*Sma*I), linearizing the plasmid with *Bbs*I, and transforming it into Y1Hgold yeast. Transformants were selected on SD/-Ura medium, with positive colonies confirmed by PCR and used to establish the stable bait strain.

Following the Matchmaker Gold One-hybrid System protocol, the bait yeast strain Y1Hgold[pABAi-*SlANS*pro] was first made competent. This was followed by separate transformations of the recombinant pGADT7-*SlMYB76* vector and the empty *pGADT7* control into the competent cells. The transformation mixtures were then spread onto SD/-Leu and SD/-Leu/ABA plates and incubated at 30 °C for 3 days, after which colony growth was observed.

### 2.8. Yeast Two-Hybrid Assay

The coding sequences (CDS) of *SlMYB76* and *SlJAF13* were inserted into the pGBKT7 (bait; *EcoR*I/*PST*I) and pGADT7 (prey; *EcoR*I/*BamH*I) vectors, respectively. To investigate their interaction, the Matchmaker Gold Yeast Two-Hybrid System was employed. Four combinations of co-transformations were set up: pGBKT7-53 with pGADT7-T, pGBKT7-*SlMYB76* with pGADT7-Empty, pGBKT7-Empty with pGADT7-*SlJAF13*, and pGBKT7-*SlMYB76* with pGADT7-*SlJAF13*. The recombinant expression vectors of these combinations were co-transferred into yeast Y2Hgold competent cells, and 100 μL of the transformation mixture was spread onto SD/−Leu/−Trp (DDO) and SD/−Ade/−His/−Leu/−Trp/+AbA/X−α−Gal (QDO/X/A) media for cultivation. After 3 days of inverted cultivation at 30 °C, the growth of yeast colonies was observed.

### 2.9. Bimolecular Fluorescence Complementation (BiFC) Assay

The coding sequences of *SlMYB76* and *SlJAF13* were directionally cloned into the pSPYNE and pSPYCE vectors, respectively, via *Sac*I and *Sal*I restriction sites. The recombinant vectors were introduced into *Agrobacterium tumefaciens* GV3101 and transiently expressed in tobacco leaves through infiltration. Imaging was performed with a confocal laser scanning microscope 48–72 h post-infiltration.

### 2.10. Split Luciferase Complementation Imaging (LCI) Assay

The coding sequences of *SlMYB76* and *SlJAF13* were cloned into the pCAMBIA1300-nLUC (*BamH*I/*Sal*I) and pCAMBIA1300-cLUC (*BamH*I/*PST*I) vectors, respectively. These constructs were then transformed into *Agrobacterium* GV3101. Tobacco plants aged between 40 and 50 days were selected for *Agrobacterium* transformation. Four co-expression combinations were established: pCAMBIA1300-nLUC with pCAMBIA1300-cLUC, *SlMYB76*-nLUC with pCAMBIA1300-cLUC, pCAMBIA1300-nLUC with *SlJAF13*-cLUC, and *SlMYB76*-nLUC with *SlJAF13*-cLUC. Each set of *Agrobacterium* cultures was injected into tobacco plants. The tobacco samples were placed in a light-free incubator for 36–48 h, after which the tobacco leaves were removed and placed on agar plates. Following substrate application, leaves were incubated in the dark for 2–5 min prior to image acquisition and analysis using a plant imaging system.

### 2.11. Co-Immunoprecipitation (Co-IP) Assay

The coding sequences of *SlMYB76* and *SlJAF13* were cloned into the pCAMBIA1300-FLAG (*BamH*I/*Sal*I) and pCAMBIA1300-GFP (*Kan*I/*Xba*I) vectors, respectively. These constructs were separately introduced into *Agrobacterium* GV3101 and used for tobacco leaf infiltration.

Following injection, the tobacco leaves were frozen using liquid nitrogen and subsequently ground to extract and purify the proteins. The final protein solution was mixed with 40 μL of sample buffer, denatured at 100 °C for 5 min, and centrifuged prior to Western blot analysis. For Western blot analysis, an appropriate volume of the supernatant was utilized, and commercial FLAG and GFP tag antibodies were employed for detection purposes.

## 3. Results

### 3.1. Screening for Genes Involved in the Differential Fruit Peel Coloring of ‘Black Pearl’ Tomato

**Figure 1:**
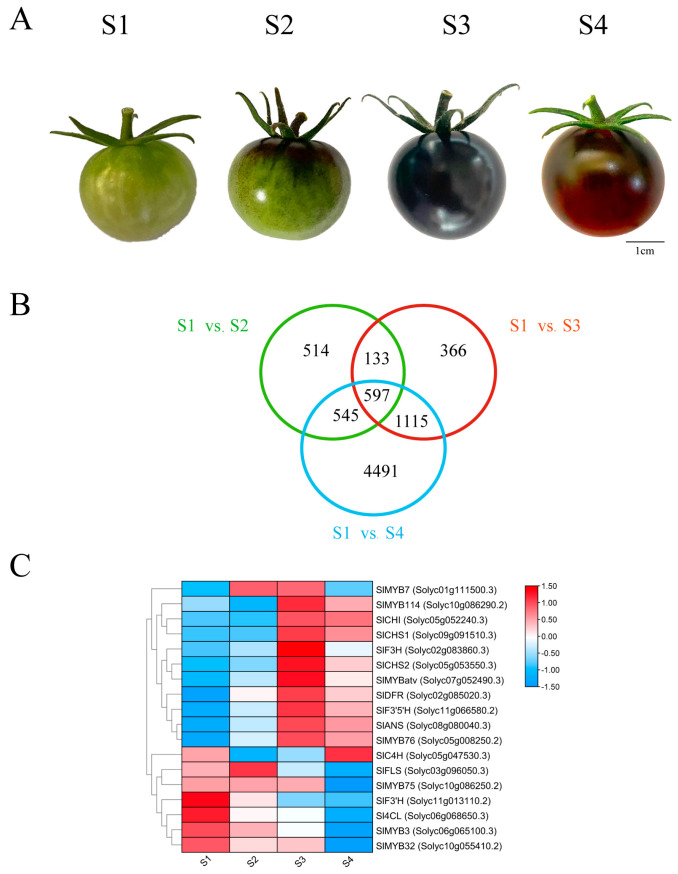
Transcriptomic analysis of fruit coloration in the ‘Black pearl’ tomato. (**A**) Fruit phenotypes at four developmental stages: S1 (mature green), S2 (coloring), S3 (purple immature), and S4 (fully ripened). Scale bar = 0.5 cm; (**B**) Venn diagram of differentially expressed genes (DEGs) across stages. (**C**) Expression patterns of key anthocyanin biosynthesis genes.

The Illumina HiSeq platform was utilized to analyze anthocyanin synthesis-related genes in the peel of the ‘Black pearl’ tomato. Sequencing was conducted at four developmental stages: mature green (S1), coloring (S2), purple immature (S3), and fully ripened (S4) (Figure 1A). A large set of differentially expressed genes (DEGs) was detected across all stages, with specific gene sets being revealed through an analysis of overlaps between different stage comparisons (Figure 1B). The number of common DEGs was 730 for S1 vs. S2 & S1 vs.S3, 1142 for S1 vs. S2 & S1 vs. S4, and 1172 for S1 vs. S3 & S1 vs. S4. From these overlaps, a core set of 597 DEGs was specifically identified during the anthocyanin-rich stages (S3 and S4), indicating their potential pivotal role in anthocyanin biosynthesis during fruit ripening.

Screening for anthocyanin pathway-related differentially expressed genes revealed distinct expression dynamics (Figure 1C). On the other hand, early genes such as *Sl4CL* and *SlC4H* showed inconsistent expression patterns during development. The expression of early biosynthetic genes, including *SlCHI*, *SlCHS1*, and *SlCHS2*, was characterized by a progressive increase during fruit development, peaking at the S3 stage before a slight decline was observed at S4. In contrast, the late biosynthetic genes (*SlF3′H*, *SlF3H*, *SlF3′5′H*, *SlDFR*, and *SlANS*) were markedly induced, exhibiting pronounced developmental variation. Their expression was slightly reduced at the S2 stage relative to the S1 stage, reached a maximum at the S3 stage, and then diminished to varying degrees by the S4 stage. Analysis of MYB transcription factors revealed distinct expression dynamics. *SlMYB7*, *SlMYB76*, and *SlMYBatv* expression correlated positively with anthocyanin accumulation, rising to a maximum at S3 stage. Conversely, *SlMYB3* and *SlMYB32* expression decreased steadily throughout fruit development, reaching minimal levels at S4 stages (Figure 1C).

**Figure 2:**
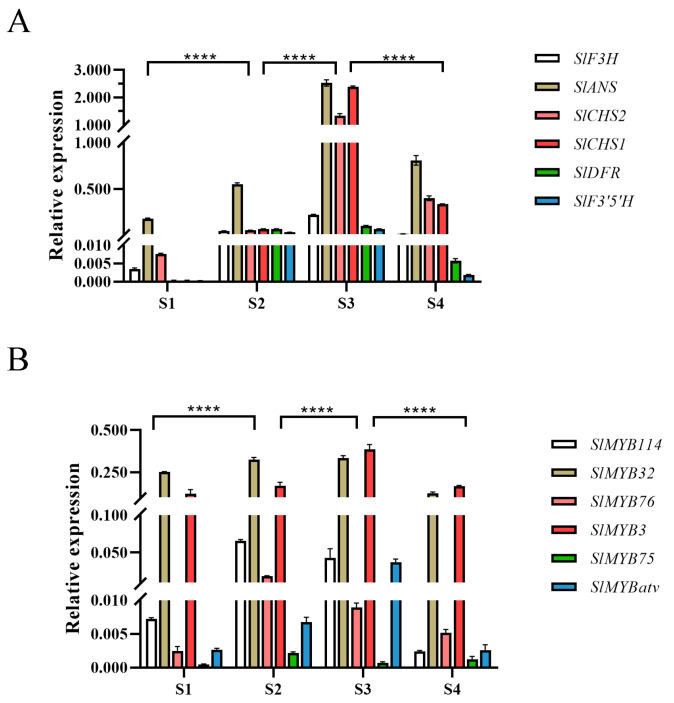
qRT-PCR validation of anthocyanin-related gene expression. (**A**) Relative expression levels of anthocyanin structural genes. (**B**) Relative expression levels of MYB transcription factors. Data are shown as mean ± SD (*n* = 3). Significant differences were determined by two-way ANOVA with Tukey’s test (**** *p* < 0.0001).

The expression dynamics of key structural genes were consistent between qRT-PCR and RNA-seq analyses. Their transcript levels increased gradually during fruit development, peaked at the S3 stage, and then slightly decreased at S4 (Figure 2A). The expression of *SlMYB114*, *SlMYB75* and *SlMYB76* peaked at the S2 stage and thereafter gradually decreased, reaching its lowest level at the S4 stage. Conversely, the transcript levels of *SlMYB3*, *SlMYB32* and *SlMYBatv* increased during the early stages of fruit development, attained their maximum at S3 stage, and then declined (Figure 2B).

Conversely, the transcript levels of *SlMYB3*, *SlMYB32*, and *SlMYBatv* were found to increase during early development, reach a maximum at S3, and subsequently decline. In contrast to the significant fluctuations of *SlMYB76* and *SlMYBatv*, the expression levels of *SlMYB3* and *SlMYB32* across developmental stages were not statistically significant. Given that *SlMYB114* (a positive regulator) and *SlMYBatv* (a negative regulator) have established roles in anthocyanin accumulation [20], and based on the present expression profiles, the investigation was subsequently focused on *SlMYB76*.

### 3.2. SlMYB76 Negatively Regulates SlANS Expression and Affects Anthocyanin Biosynthesis in the Peel of the ‘Black Pearl’ Tomato

**Figure 3:**
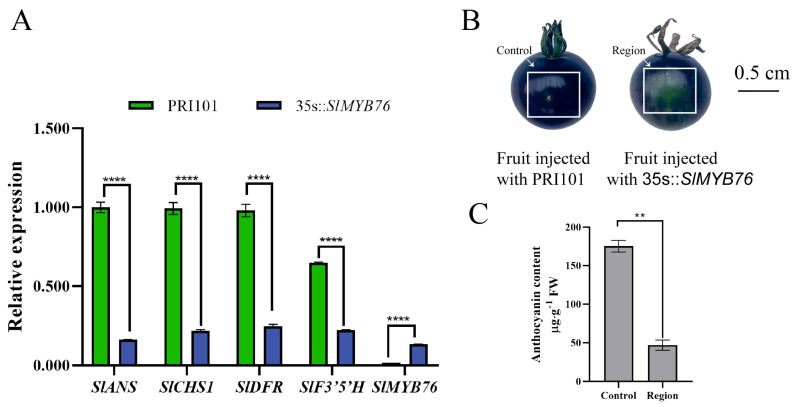
*SlMYB76* overexpression represses anthocyanin production. (**A**) Expression analysis of *SlMYB76* and its target genes. Data are mean ± SD (*n* = 3); **** p < 0.0001 (two-way ANOVA, Sidak’s test). (**B**) Representative fruit peel phenotypes. “Control”: area infiltrated with empty vector (pRl101); “Region”: area infiltrated with pRl101-*SlMYB76*. Scale bar = 0.5 cm. (**C**) Anthocyanin content in control and overexpression regions. Data are mean ± SD (*n* = 3); ** *p* < 0.01 (Student’s *t*-test).

To functionally characterize *SlMYB76*, transient overexpression lines were generated. The peel of the transformed fruits exhibited a significantly lighter coloration compared to the empty vector control (Figure 3B), concomitant with a marked reduction in anthocyanin content (Figure 3C). Expression profiling in these lines revealed significant downregulation of key anthocyanin biosynthetic genes, including *SlANS*, *SlCHS1*, *SlDFR*, and *SlF3′5′H*, with the most pronounced suppression being observed for *SlANS* (Figure 3A). These collective findings are consistent with a role for *SlMYB76* as a negative regulator of anthocyanin biosynthesis, primarily achieved through the repression of *SlANS* expression.

**Figure 4:**
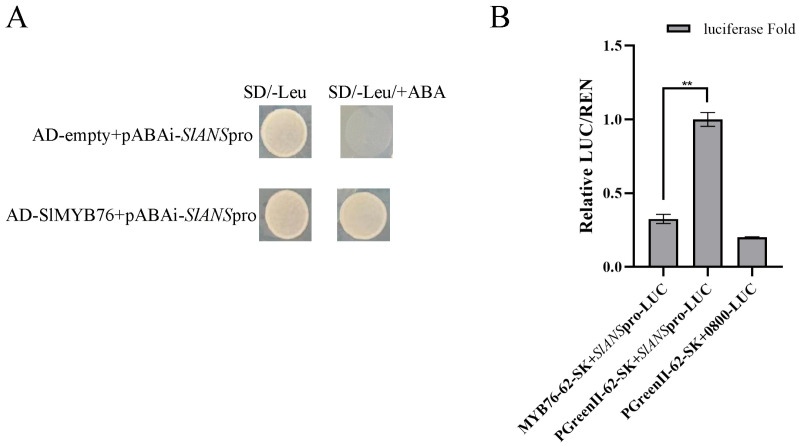
SlMYB76 directly represses *SlANS* transcription. (**A**) Yeast one-hybrid assay showing SlMYB76 binding to the *SlANS* promoter; (**B**) dual-luciferase assay confirming SlMYB76*−*mediated repression of *SlANS* promoter activity. Data are mean ± SD (*n* = 3); ** p < 0.01 (Student’s *t*-test).

A direct molecular interaction between *SlMYB76* and the *SlANS* promoter was first confirmed in vitro by a yeast one-hybrid assay (Figure 4A). Subsequent *in planta* analysis via a dual-luciferase transient expression system functionally validated the repressive nature of this binding. Specifically, a significant reduction in luminescent signal was recorded in tobacco leaves when *SlMYB76* was co-infiltrated with the *ProSlANS::LUC* construct, compared to the control. This suppression of promoter activity provides direct mechanistic evidence that *SlMYB76* functions as a transcriptional repressor of *SlANS*, explaining its downregulation in overexpression studies (Figure 4B).

### 3.3. The Interaction Between the Tomato SlMYB76 Transcription Factor and the bHLH Transcription Factor SlJAF13

**Figure 5:**
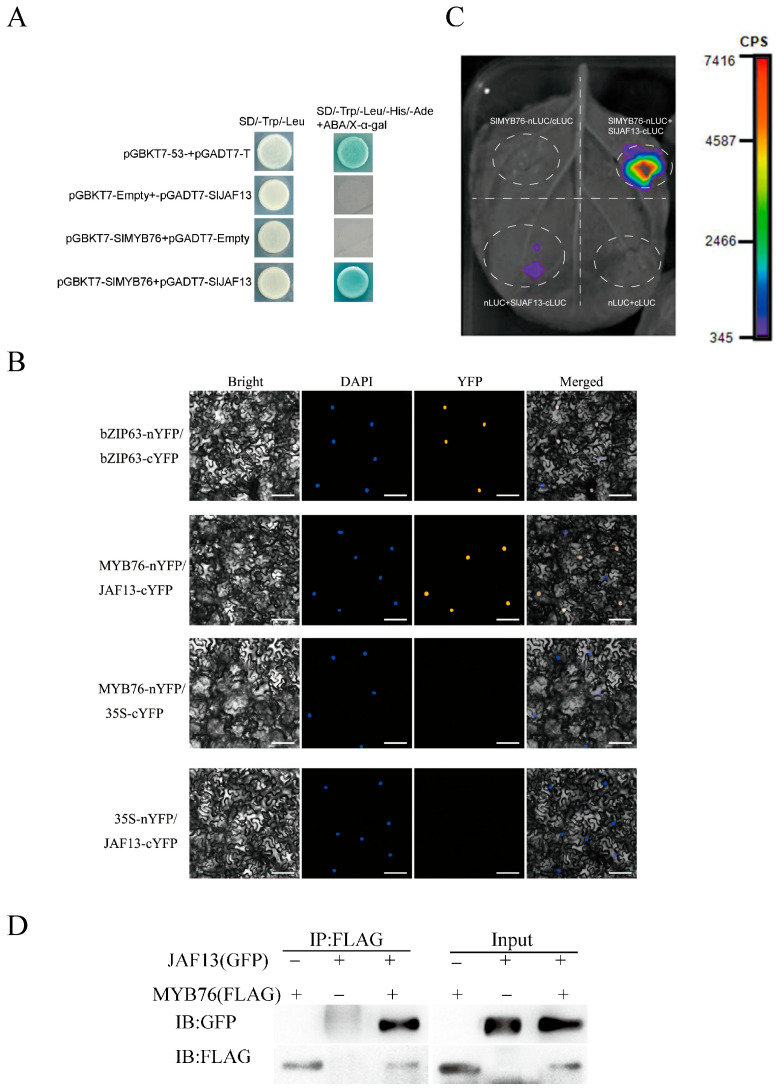
SlMYB76 interacts with SlJAF13. (**A**) Protein–protein interaction analyzed by yeast two-hybrid assay on selective media. (**B**) Bimolecular fluorescence complementation (BiFC) showing nuclear localization of the complex in tobacco epidermal cells. Scale bar = 25 μm. (**C**) In vivo interaction detected by split-luciferase complementation imaging (LCI). (**D**) Physical association confirmed by co-immunoprecipitation (Co-IP) using tobacco protein extracts.

The potential formation of a repressive complex between SlMYB76 and SlJAF13 was investigated through a combination of in vivo and in vitro experiments. The conserved region sequence of the *SlMYB76* was used as the bait, and different experimental and control groups were established. The yeast two-hybrid assay results revealed that the yeast colonies in the positive control groups, negative control groups, and experimental groups were able to grow normally on SD/−Trp/−Leu medium without ABA and X–α–gal. However, only the positive control group and experimental group were able to grow and turn blue on SD/−Trp/−Leu/−His/−Ade medium containing ABA and X–α–gal (Figure 5A). This indicates that SlMYB76 and SlJAF13 interact with each other in yeast.

Transient expression assays in tobacco involved SlMYB76-nYFP and SlJAF13-cYFP, together with positive and negative control combinations. The specific interaction between SlMYB76-nYFP and SlJAF13-cYFP was demonstrated by yellow fluorescence signals localized in the nucleus, a pattern comparable to the positive control. However, no fluorescence signals were observed in the other control groups (Figure 5B). Based on the bimolecular fluorescence complementation (BiFc) assay, an interaction between the transcription factor SlMYB76 and SlJAF13 was demonstrated in the nucleus. To this end, the pCAMBIA1300-SlMYB76-nLUC and pCAMBIA1300-SlJAF13-cLUC vectors were constructed via Gateway technology for further in vivo validation.

For transient expression in tobacco, the recombinant vectors were introduced into *Agrobacterium* GV3101 and infiltrated into fully expanded leaves. Detectable luminescence in the split-luciferase complementation imaging (LCI) assay was observed only upon co*–*expression of *SlMYB76–*nLUC and *SlJAF13–*cLUC (Figure 5C), while all control combinations remained non*–*luminescent. This result confirmed the specific interaction between *SlMYB76* and *SlJAF13 * in vivo.

Additionally, immunoprecipitation (Co–IP) was performed using FLAG and GFP antibodies. The successful co–transformation was confirmed by the presence of clear bands in the input samples. The interaction between SlMYB76 and SlJAF13 in tobacco was further validated through the detection of the products via western blotting (Figure 5D). The results presented herein collectively demonstrate a probability of protein–protein interaction between the SlMYB76 transcription factor and the bHLH transcription factor SlJAF13. However, the detailed mechanism underlying the formation of the SlMYB76–SlJAF13 protein complex and their coordinated regulation of *SlANS* gene expression requires further verification.

### 3.4. Metabolomic Analysis of ‘Black Pearl’ Tomato Fruit Color Formation

**Figure 6:**
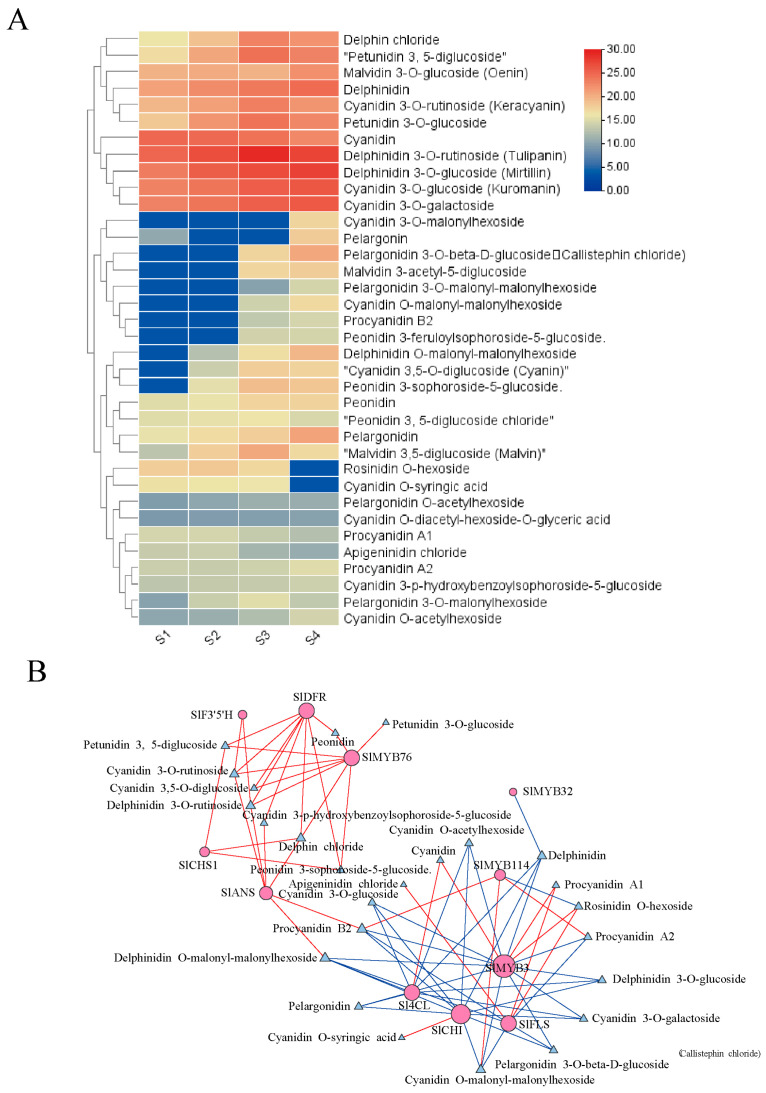
Selection of differentially metabolite related to fruit coloration in the ‘Black pearl’ tomato: (**A**) Differential metabolite clustering analysis; (**B**) network analysis of differential genes and differential metabolites.

A non-targeted metabolomic analysis was performed to profile the metabolic changes during color transition, leading to the identification of 36 metabolites across the S1 to S4 stages (Figure 6A). Cyanidin and pelargonidin derivatives were characterized as the principal pigments in the ‘Black Pearl’ peel. Significant accumulation during development was observed for several derivatives, including cyanidin-3-O-glucoside, cyanidin-3-O-rutinoside, and pelargonidin-3-O-glucoside. In contrast, delphinidin-3-O-glucoside and petunidin-3-O-glucoside remained stable, and pelargonidin levels were minimal at early stages. The close alignment of their abundance patterns with the total anthocyanin content confirmed their role as the major colorants.

Correlation network analysis, integrating transcriptomic and metabolomic datasets, revealed a tightly linked module comprising key anthocyanin structural genes (*SlCHS1*, *SlF3′5′H*, *SlDFR*, *SlANS*), the regulatory repressor SlMYB76, and pivotal flavonoid metabolites, including luteolin-3-O-rutinoside and cyanidin-3-O-rutinoside (Figure 6B). This coordinated pattern implies that the expression of these genes orchestrates the metabolic reprogramming underlying color change. *SlMYB76* may function to fine-tune the accumulation of anthocyanin-pathway intermediates and end-products bytranscriptionally suppressing late biosynthetic genes. This regulatory action could account for the net negative effect on anthocyanin synthesis observed in our functional studies. A central finding from this integrative analysis is the identification of *SlANS* as a potential critical bottleneck enzyme. Its strong correlation with both flavonoid and anthocyanin metabolites suggests its potential role as a key junction point, channeling the flavonoid metabolic flow into anthocyanin production, and thereby providing aplausible link between core flavonoid metabolism the specific pigmentation phenotype in this system.

## 4. Discussion

Anthocyanins are a class of water-soluble pigments that impart the characteristic red, purple, and blue hues to numerous plant tissues, including fruits, flowers, and leaves. Anthocyanin pigmentation serves as a visible indicator of plant physiological status, with its accumulation representing a sophisticated adaptation mechanism integral to both developmental processes and stress responses. The anthocyanin biosynthetic pathway is under the control of a complex transcriptional regulatory network. One key regulator is the MBW complex, with MYB transcription factors being the major players in this complex. In tomato, several MYB transcription factors have been investigated for their involvement in anthocyanin synthesis. In the ‘Black Pearl’ tomato, anthocyanins primarily accumulate in the peel. Although there is close substance transport and signal coordination between the peel and flesh, we found that the peel and flesh exhibit different coloration patterns at various developmental stages. The flesh remained green without visible color changes from the S1 to S3, and turns red at the S4 stage. Therefore, the mechanisms underlying color formation differ between the peel and flesh. This study focuses on the mechanism of color formation in the peel, and the peel was selected as the experimental material. In future research, we will further explore the molecular mechanisms of color formation in tissues such as the flesh, as well as the coordinated regulatory mechanisms among different tissues.

In this study, the investigation was centered on four specific MYB transcription factors: *SlMYB114*, *SlMYB76*, *SlMYB3*, and *SlMYB32*, with the latter being identified as a homolog of *AtMYB32* (Figure 1C). The expression patterns of these genes were examined throughout tomato peel development, and their potential roles in the regulation of anthocyanin synthesis were investigated. The results showed that all four genes were expressed in tomato fruits, albeit with different expression patterns during fruit development. *SlMYB114* and *SlMYB75* belonged to the SG6 subgroup, which is known to promote anthocyanin synthesis. *SlMYB114* has previously been established as a positive regulator of anthocyanin synthesis and accumulation in tomato. By using CRISPR/Cas9 technology to create a mutant similar to *SlAN2*, a gene involved in anthocyanin biosynthesis, researchers observed changes in multiple genes expression related to anthocyanin production [18]. On the other hand, *SlMYB76*, *SlMYB3*, and *SlMYB32* were classified under the SG4 subgroup, which inhibits anthocyanin synthesis.

Phylogenetic analysis of SlMYB76 with *Arabidopsis* SG4 members (*AtMYB4*, *AtMYB3*, *AtMYB32*, and *AtMYB7*) confirmed that *SlMYB76* is associated with anthocyanin biosynthesis and clusters within the SG4 subgroup (Figure S1). Further clustering analysis with SG4 MYBs from diverse plant families revealed that *SlMYB76* belongs to the SG4-*FaMYB1* subclass, whose members are characterized as negative regulators of anthocyanin biosynthesis (Figure S2). Within the evolutionary tree of SG4 MYB repressors identified in tomato, *SlMYB76* first clusters with homologs from *Petunia hybrida* and *Vitis vinifera*, and then with *FaMYB1*, indicating closer evolutionary relationships and potentially conserved functions among these genes. Consistent with other *FaMYB1*-like R2R3-MYB repressors, which act as co-repressors integrated into or bound to the MBW (MYB-bHLH-WD40) complex to suppress target genes, *SlMYB76* likely participates in multi-level inhibition of anthocyanin biosynthesis, including repressing late structural genes and limiting activator abundance, as observed for *FaMYB1*-like repressors in petunia, grape, and poplar. In contrast, *AtMYB4*-subclass repressors (*PtoMYB156*, *AtMYB3*, *AtMYB32*, *AtMYB7*) employ an active repression mechanism by competing with activating MYBs for binding sites in downstream structural gene promoters [21]. *SlMYB76* diverges from this mode: in tomato, its repressive function is mediated through direct interaction with SlJAF13 (a bHLH partner), thereby suppressing the expression of the late structural gene *SlANS*. This comparative analysis highlights that *SlMYB76* represents a distinct SG4-*FaMYB1*-like repressor in tomato, utilizing a bHLH-interaction-dependent mechanism to modulate anthocyanin biosynthesis, which enriches the functional diversity of SG4 MYB repressors in plants.

As shown in Figure S3, these *SlMYB* genes all contain a conserved bHLH-interacting domain, consistent with their classification as typical repressive R2R3-MYB transcription factors belonging to subgroup 4 (SG4). The R2R3 domains, which are responsible for specific DNA binding to target promoters and interactions with cofactors, are highly conserved across these homologs [22]. Among the aligned sequences, most tomato SG4 MYB repressors harbor an EAR motif, except for *AtMYB3* (from *Arabidopsis*) and *CaMYB101* (from *Capsicum*), which lack this domain. Additionally, a second repressive motif, TLLLFR (designated as C5), was identified in the C-terminal region of these *FaMYB1*-like repressors. This is consistent with findings in *Populus*, where *PtrMYB182* exerts its repressive function primarily through the TLLLFR motif rather than the EAR domain, suggesting a conserved alternative repression mechanism among *FaMYB1*-like factors across plant genera, including tomato [23]. These comparisons highlight that *SlMYB76* shares core structural features with other tomato SG4 MYB repressors (e.g., conserved bHLH-interacting and R2R3 domains) while exhibiting functional parallels with *FaMYB1*-like factors in utilizing non-EAR motifs (e.g., TLLLFR) for repression, enriching our understanding of MYB-mediated regulation in tomato species [21].

The dramatic color shift from green to purple-red in ‘Black Pearl’ peel was quantitatively associated with the accumulation of specific anthocyanin species (Figure 1A and Figure 6A). Our analysis identified cyanidin- and pelargonidin-derived pigments, rather than delphinidin or petunidin derivatives, as the principal contributors to the characteristic coloration. A correlative link was established between the transcriptional levels of key biosynthetic genes (*SlCHS1*, *SlF3′5′H*, *SlDFR*, *SlANS*) and the accumulation of specific metabolites including cyanidin-3-O-rutinoside (Figure 6A,B). Notably, *SlMYB76* expression is also correlated with these metabolites, likely an indirect effect of its co-regulation of the structural genes. This metabolite signature shares similarities with other anthocyanin-rich fruits like jujube and red-skinned pears [24,25], suggesting convergent evolutionary pathways for color development. To further elucidate the functional role of *SlMYB76*, future studies will be performed, including gene knockout or silencing experiments. These functional assays are expected to directly link *SlMYB76* activity to metabolite accumulation, thereby confirming its causal relationship and refining its regulatory mechanism within the biosynthesis pathway.

Direct evidence for the repressive function of *SlMYB76* was provided by transient overexpression assays, wherein elevated expression was shown to cause significant effects at both the phenotypic and transcriptional levels, manifesting as reduced anthocyanin accumulation and downregulation of key biosynthetic genes, particularly *SlANS* (Figure 3). Consequently, *SlMYB76* was positioned within the negative regulatory circuitry of anthocyanin biosynthesis. Notably, its expression peak was observed to precede maximal pigment accumulation (S2 vs. S3; Figure 2B). A dual-phase regulatory model is therefore proposed: the early expression peak may establish a threshold to prevent premature synthesis, while its subsequent decline could represent a feedback mechanism for fine-tuning production.

The regulatory landscape is further complicated by interactions with bHLH partners. In tomato, SlJAF13 is a well-characterized bHLH factor that promotes anthocyanin synthesis by activating *SlAN1* and can be sequestered by the JA-signal repressor SlJAZ2. Furthermore, a competitive interaction paradigm exists, where the repressor SlMYBATV binds SlJAF13, and this interaction can be disrupted by the activator SlAN2-like, thereby de-repressing anthocyanin synthesis [21]. A comprehensive interaction analysis employing Y2H, BiFC, LCI, and Co-IP assays unequivocally confirmed the physical interaction between SlMYB76 and SlJAF13 in the nucleus (Figure 5). However, the interaction between SlMYB76 and SlJAF13 identified in this study was validated in a heterologous tobacco system. Although this system is widely used and provides reliable results, its functional confirmation within the homologous context of the tomato peel remains an important issue to be addressed in the future.

In summary, the formation of the SlMYB76-SlJAF13 complex may function to repress *SlANS*, a key gateway enzyme in the anthocyanin pathway. A mechanistic parallel is noted with the strawberry system, where anthocyanin and flavonol synthesis is suppressed by *FaMYB1* through its interaction with bHLH proteins, a repression that is reversible by competing MYB activators [26]. Beyond this mechanistic parallel, a critical question remains regarding whether SlJAF13 functions merely as a binding platform for SlMYB76 or actively contributes to the repression of *SlANS*, a point that warrants further investigation. Ultimately, a direct linkage between the transcriptional regulatory network and the phenotypic outcomes was established through metabolomic profiling.

## 5. Conclusions

A novel regulatory module was identified in which the R2R3-MYB transcription factor SlMYB76 partners with the bHLH factor SlJAF13 to form a repressive complex that is proposed to downregulate *SlANS* expression, thereby negatively modulating anthocyanin accumulation in the peel of the ‘Black Pearl’ tomato. This discovery enriches the current model of the tomato flavonoid regulatory network by introducing a crucial layer of negative control. These findings provide an enhanced understanding of flavonoid metabolism in Solanaceous crops and offer valuable genetic targets for the development of new cultivars designed to improve key agronomic traits, including color and nutritional value.

## Data Availability

The datasets generated and analyzed during the current study are available at NCBI project PRJNA967997. The other data supporting the conclusions of this article will be made available by the authors on request. Any reasonable requests are available from the corresponding author.

## Supplementary Materials

The following supporting information can be downloaded at: https://www.mdpi.com/article/10.3390/genes16111291/s1, Figure S1: Evolutionary tree analysis of tomato *R2R3-MYB* genes in fourth and sixth subgroups. Red clade represent the 4nd subgroup, blue means 6nd subgroup of *R2R3-MYB*; Figure S2: Phylogenetic and conserved motif analysis of candidate MYB and selected flavonoid related MYB regulators; Figure S3: The characteristic R2/R3 domain, bHLH binding domain, and EAR motif are shown with red frame respectively. A2box are shown with a green frame.; Table S1: The original data of the figure showing the expression patterns of key anthocyanin biosynthesis genes; Table S2: The original data of the figure showing differential metabolite clustering analysis.

## Abbreviations

The following abbreviations are used in this manuscript:

ROS	reactive oxygen species
PAL	phenylalanine ammonia-lyase
CHS	chalcone synthase
CHI	chalcone isomerase
F3H	flavanone 3-hydroxylase
DFR	dihydroflavonol 4-reductase
ANS	anthocyanidin synthase
UFGT	UDP-glucose: flavonoid 3-O-glucosyl transferase
ANR	anthocyanin reductase
LAR	leucoanthocyanidin reductase
