# Supplementary figures and images for: SlMYB76, an SlANS-Repressing R2R3-MYB Transcription Factor, Regulates Anthocyanin Accumulation in ‘Black Pearl’ Tomato (Solanum lycopersicum)
Source: Genes (Basel). 2025 Oct 30;16(11):1291. doi: 10.3390/genes16111291 (PMC12652620; doi:10.3390/genes16111291)

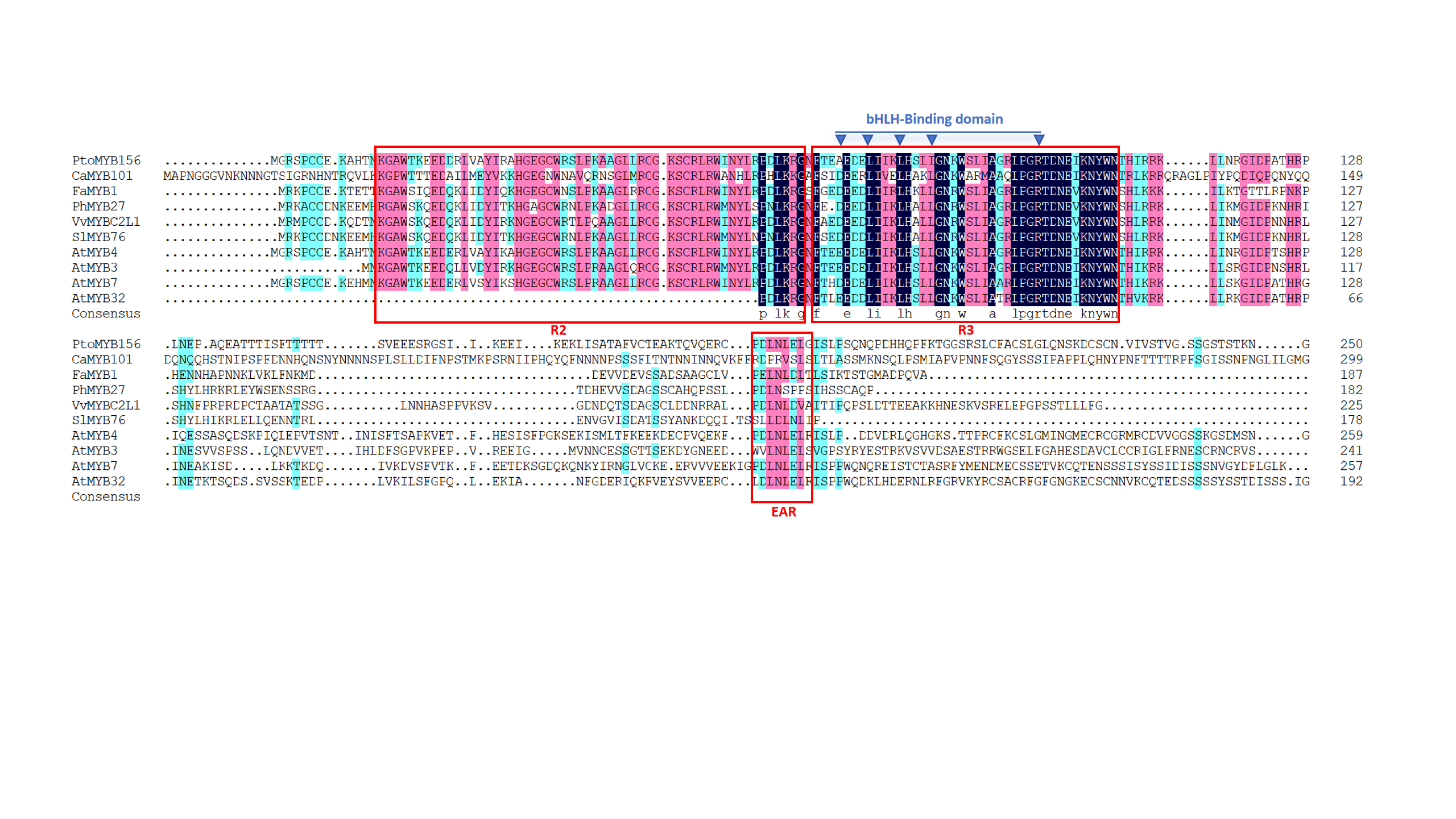

Supplement: Supplementary file 1 [file genes-16-01291-s001.zip › Figure S3.tif]

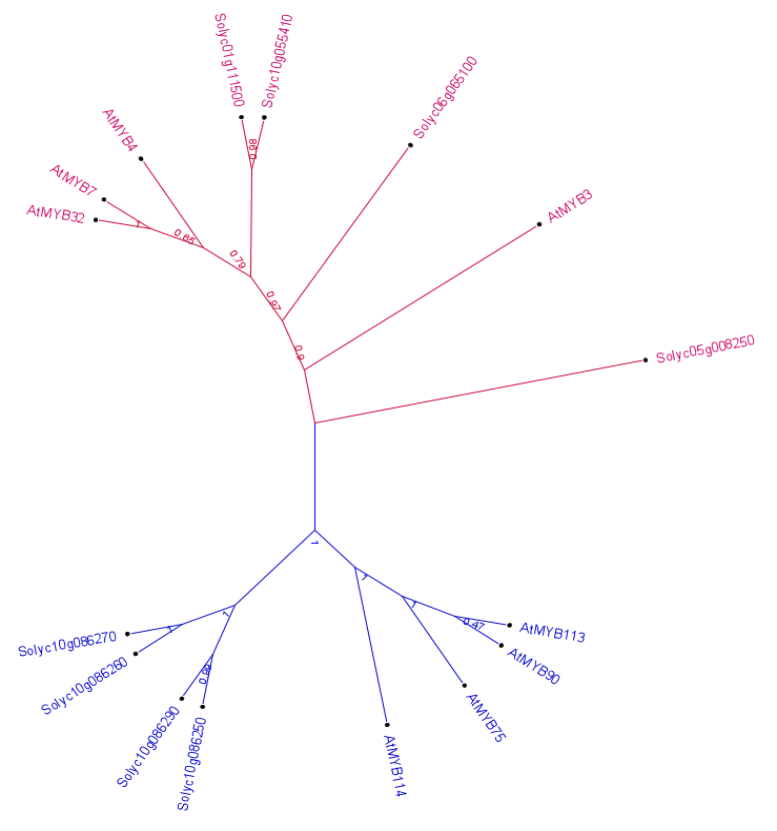

Supplement: Supplementary file 1 [file genes-16-01291-s001.zip › Figure S1.tif]

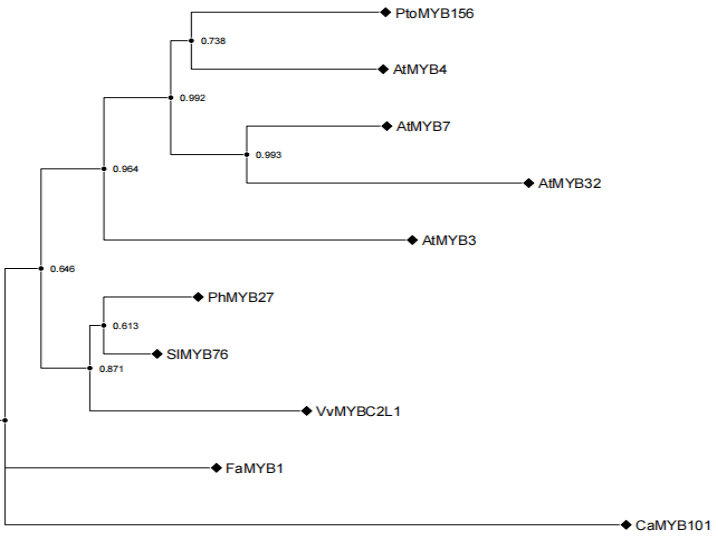

Supplement: Supplementary file 1 [file genes-16-01291-s001.zip › Figure S2.png]
